# Supplementary figures and images for: Novel viruses of the family Partitiviridae discovered in Saccharomyces cerevisiae
Source: PLoS Pathog. 2023 Jun 7;19(6):e1011418. doi: 10.1371/journal.ppat.1011418 (PMC10281585; doi:10.1371/journal.ppat.1011418)

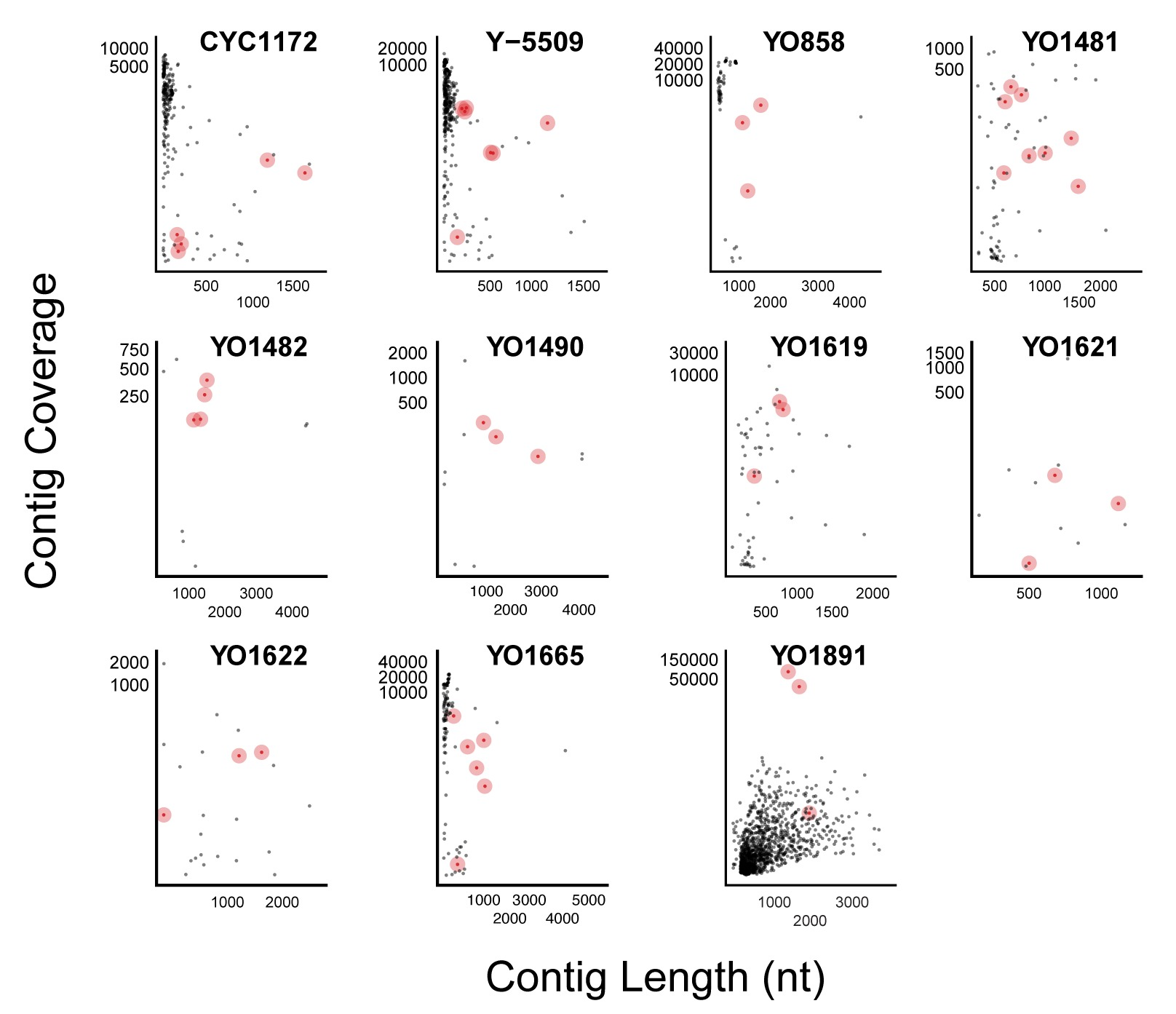

Supplement: S1 Fig — Each point represents a single contig generated by RNA sequencing. Red points are contigs that had sequence homology to known PVs by BLASTx. (TIF) [file ppat.1011418.s001.tif]

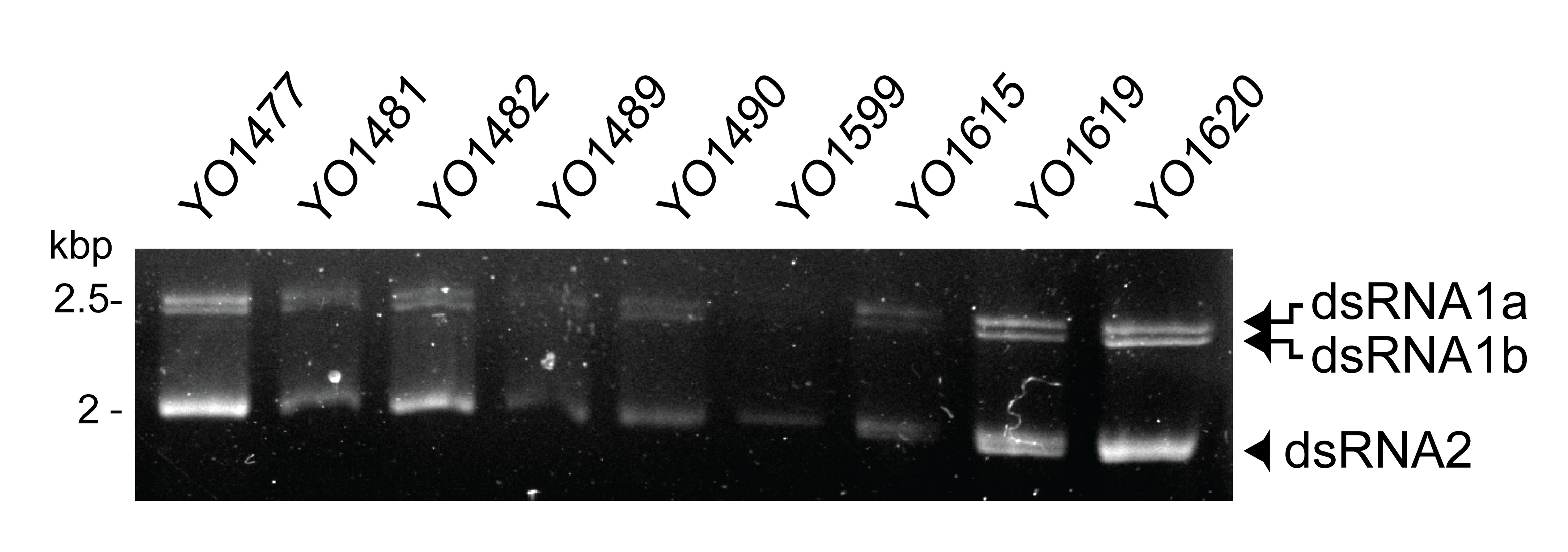

Supplement: S2 Fig — To corroborate the results of the multiplex RT-PCR screen, dsRNAs from nine strains were extracted and electrophoresed in a 3.2% agarose gel slab at 140V for 245 min and stained with 0.5 μg/mL ethidium bromide for 40 min. (TIF) [file ppat.1011418.s002.tif]

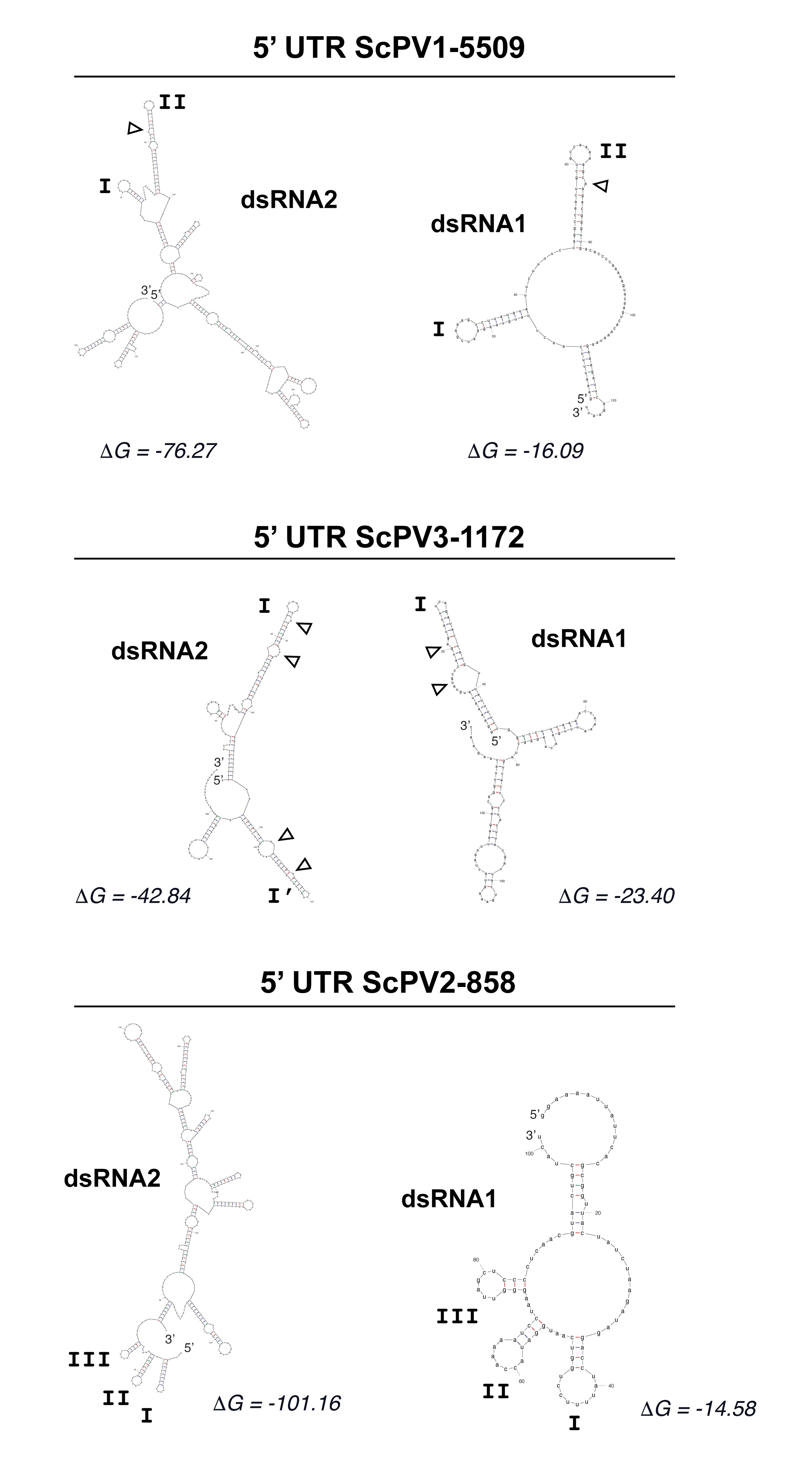

Supplement: S3 Fig — Numerals are used to denote similar stem-loop structures in the RNA, and arrows are used to mark bulges. (TIF) [file ppat.1011418.s003.tif]

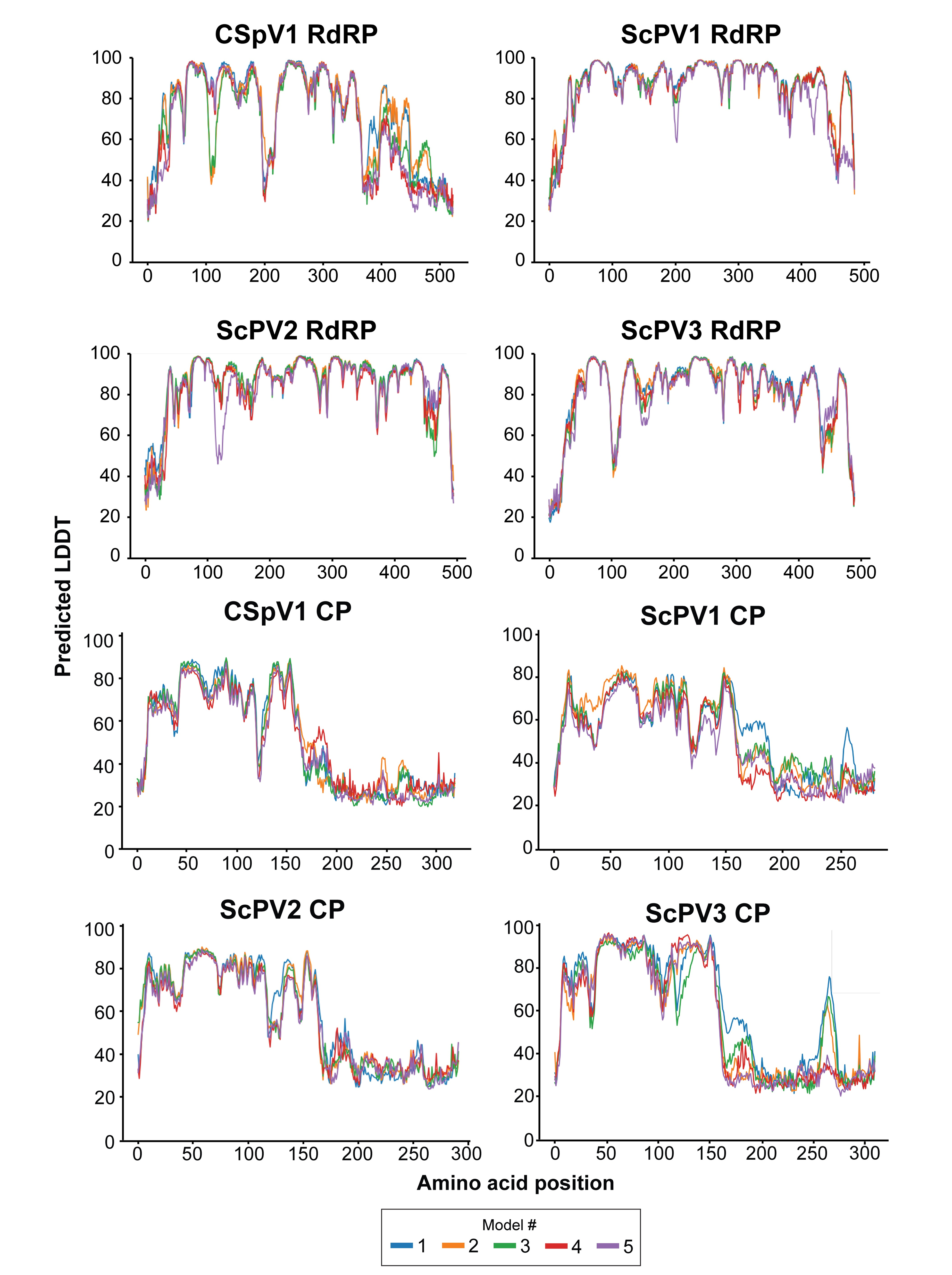

Supplement: S4 Fig — (TIF) [file ppat.1011418.s004.tif]

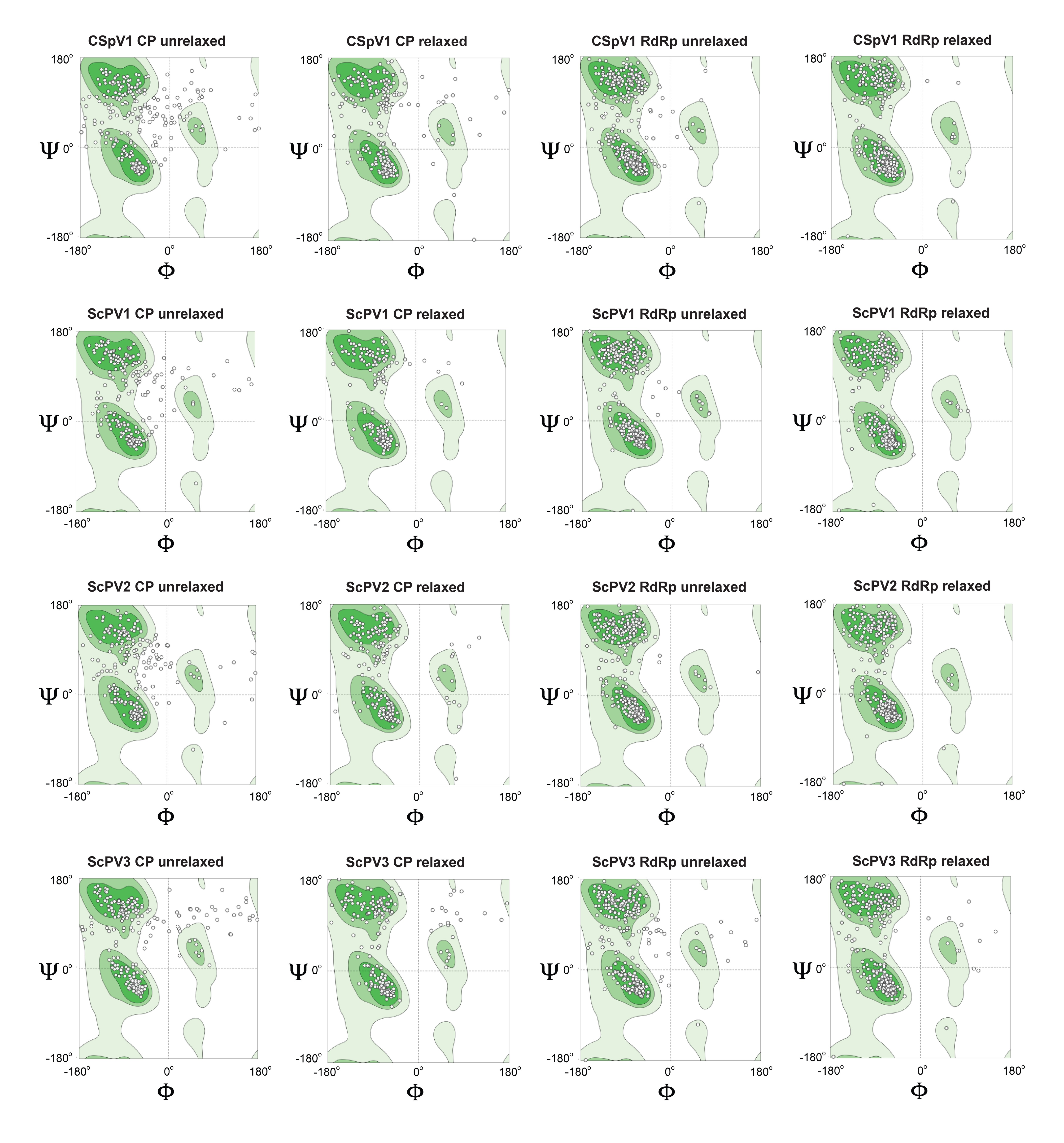

Supplement: S5 Fig — Ramachandran plots of AlphaFold2 models for CP and RdRP proteins. Each point represents an amino acid residue and its ɸ and Ѱ bond angles. The shaded area of the plots represents standard angles for α-helices, β-sheets, and left-handed helices from a database of 12,521 non-redundant experimental structures. (TIF) [file ppat.1011418.s005.tif]

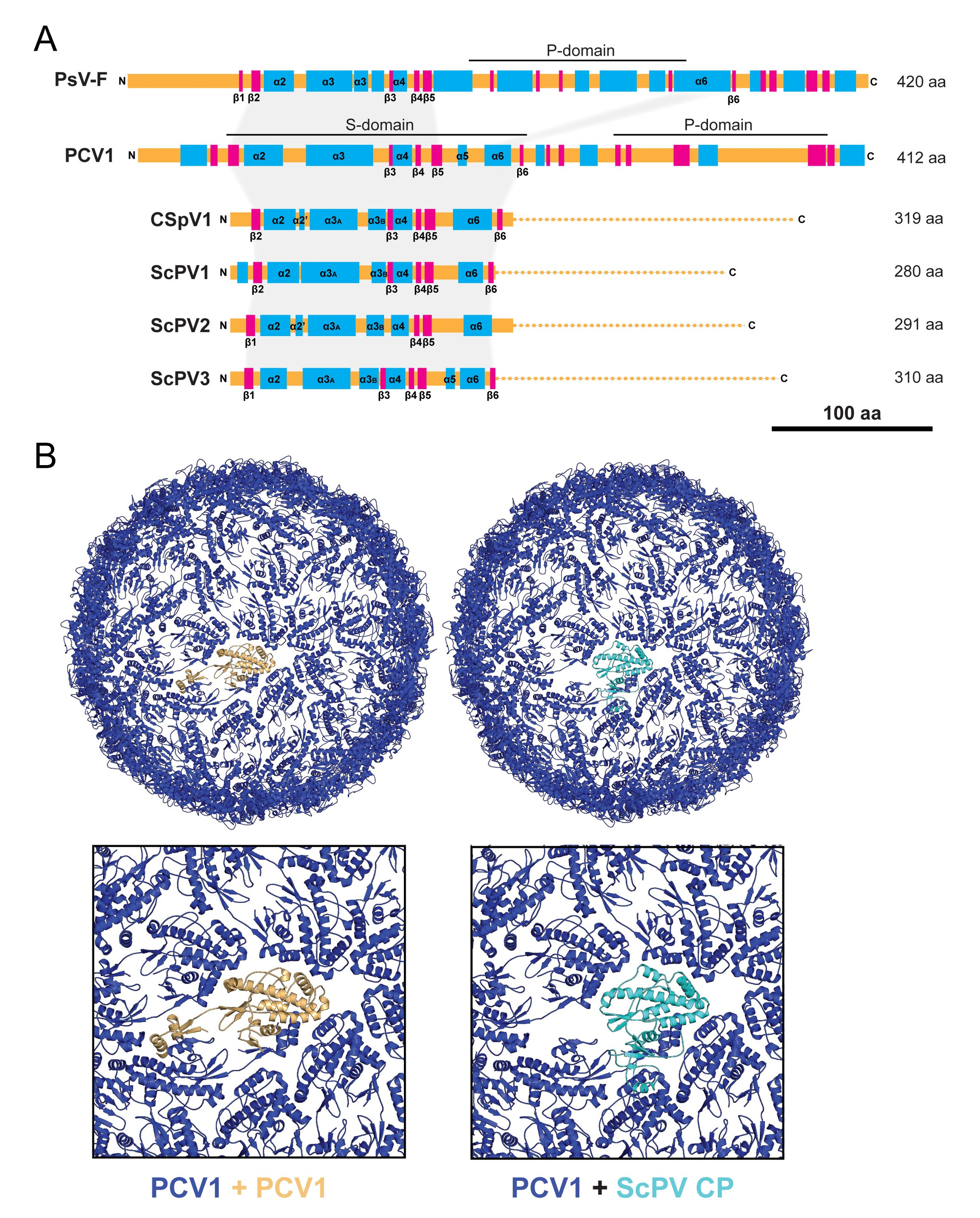

Supplement: S6 Fig — (A) Secondary structures were derived from crystal structures of the CP of PCV1 (PDB:7ncr) and PsV-F (PDB:3es5) and molecular models of ScPVs and CsPV1. The α-helices and β-sheets are represented as blue and magenta boxes, respectively. The P-domain α-helices and β-sheets are numbered relative to PCV1, with the commonalities between structures highlighted in gray. Unstructured polypeptide chains are represented in orange. The C-terminal domains of ScPVs and CsPV1 that were not modeled with high confidence are represented by dashed lines. (B) A comparison between the native PCV1 particle structure and replacing a CP monomer from PCV1 (left panel, beige) with a single CP monomer from ScPV1 (right panel, light blue). (TIF) [file ppat.1011418.s006.tif]

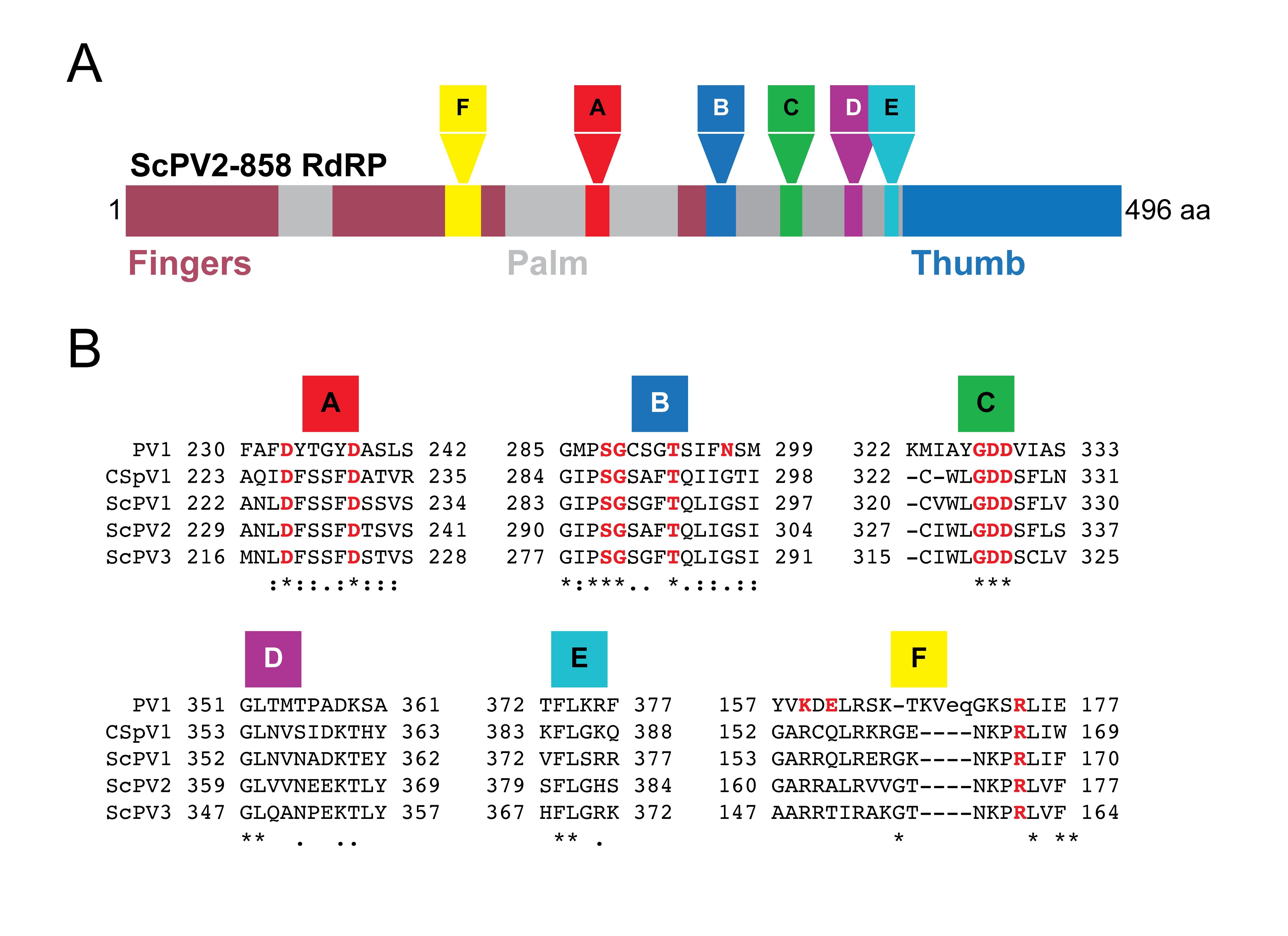

Supplement: S7 Fig — (A) Domain diagram of the RdRP of ScPV2-858 showing the position of motifs A-F. (B) A multiple sequence alignment of the residues of the RdRP conserved catalytic motifs. Red text indicates residues 100% conserved between the positive sense RNA viruses [88], CSpV1, ScPV1, ScPV2, and ScPV3. (TIF) [file ppat.1011418.s007.tif]

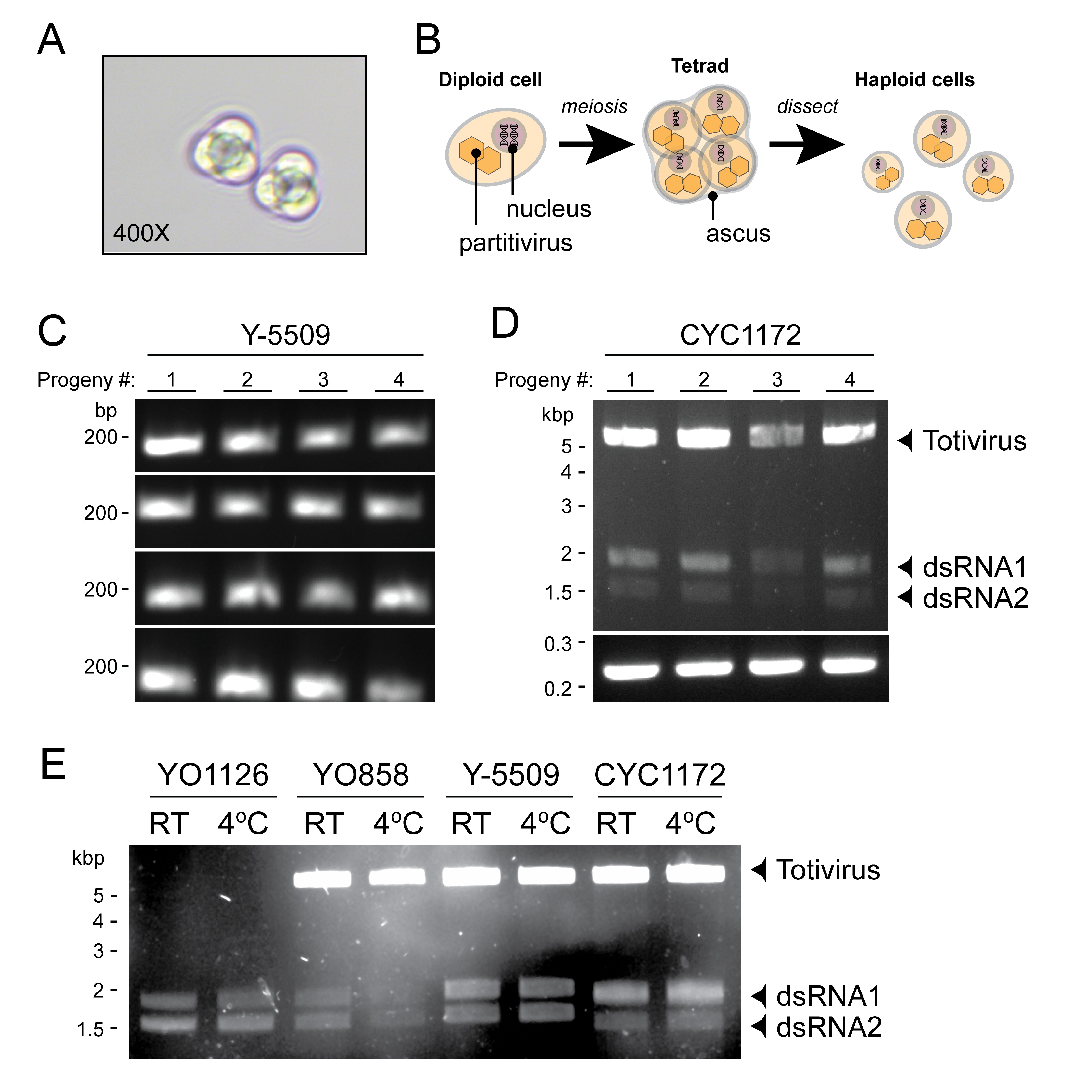

Supplement: S8 Fig — (A) A micrograph depicting S. cerevisiae asci. (B) Schematic of non-Mendelian inheritance of ScPVs during sporulation. Detection of ScPV using RT-PCR in the haploid progeny of four dissected asci of (C) Y-5509 and (D) bottom panel CYC1172. (D) Detection of ScPV3 in the haploid progeny of a dissected asci of CYC1172 by extracting dsRNAs. (E) The presence of ScPV was measured by dsRNA extraction after maintenance on agar plates incubated at either room temperature or 4°C for 6 weeks. (TIF) [file ppat.1011418.s008.tif]

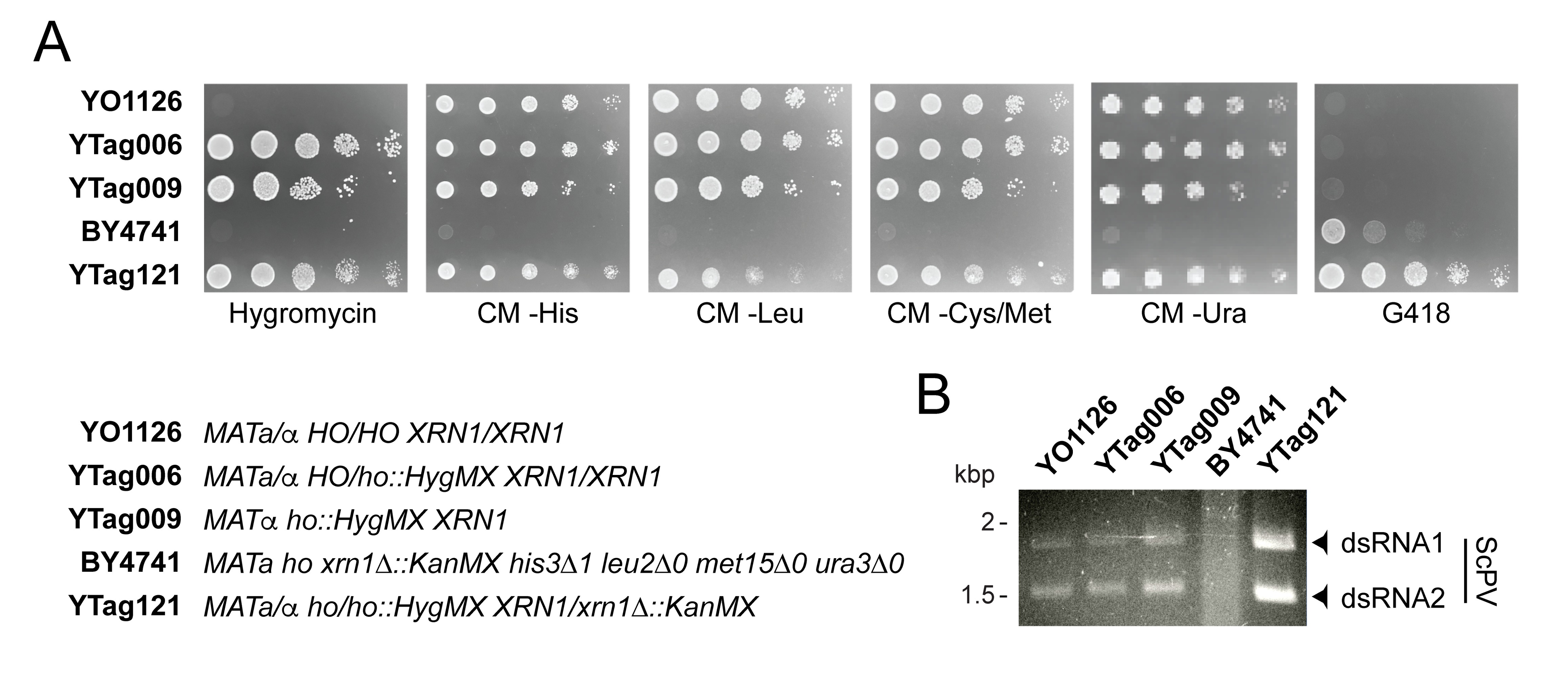

Supplement: S9 Fig — (A) Confirmation of expected genotypes using selective growth media. (B) Confirmation of the inheritance of ScPVs during strain construction by cellulose chromatography and gel electrophoresis. (TIF) [file ppat.1011418.s009.tif]

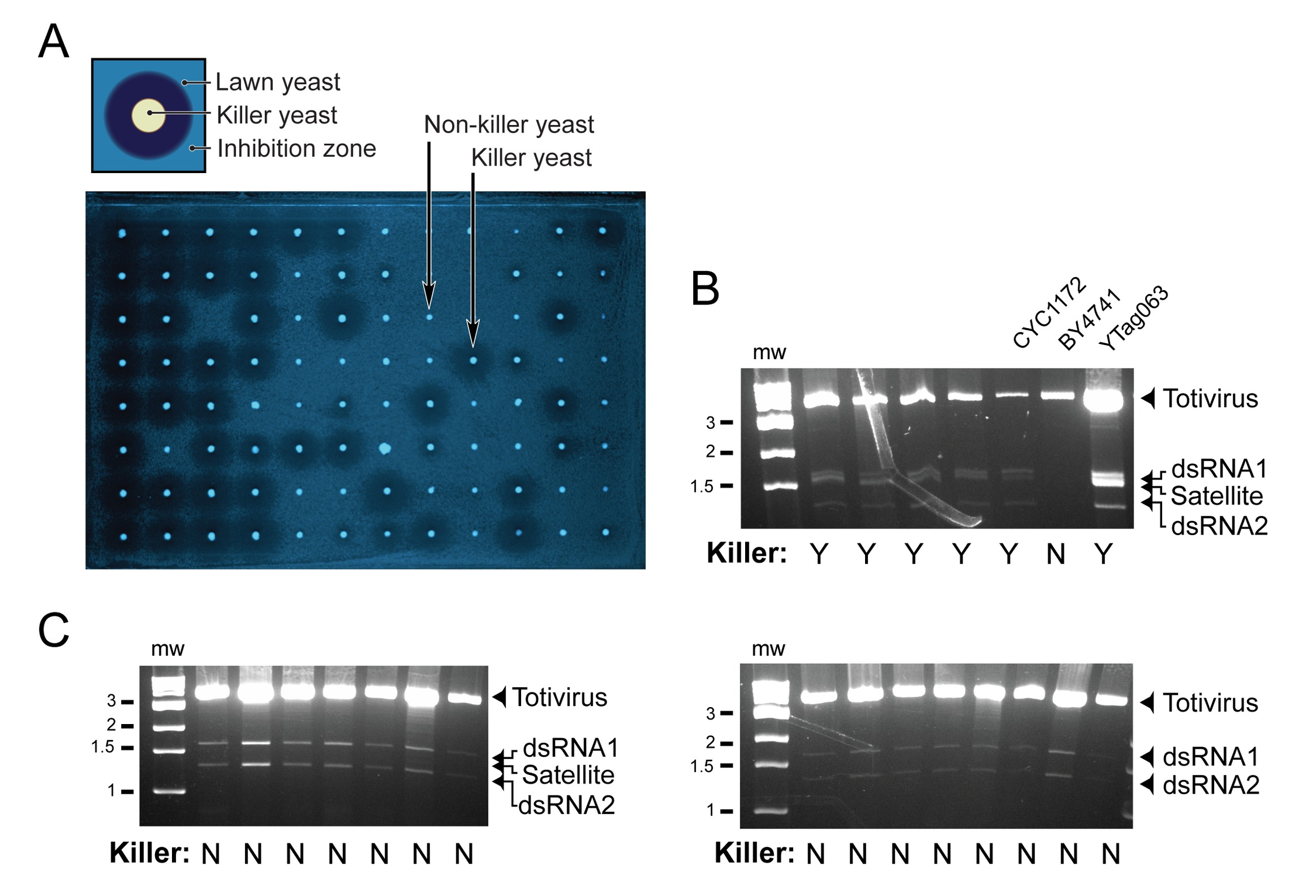

Supplement: S10 Fig — (A) Curing of the M2 satellite dsRNA from YTag085 by XRN1 expression as assayed by an agar plate killer assay (B) Extraction of dsRNAs from killer yeasts isolated from the plate in panel A. CYC1172 and YTag063 (killer yeast strains) and BY4741 (non-killer yeast strain) were included as controls for the presence or absence of a satellite dsRNA. (C) Loss of M2 and presence of ScPV3-1172 from representative non-killer strains isolated from the plate in panel A. (TIF) [file ppat.1011418.s010.tif]
